# M. Blandin’s Case of Exophthalmia, &c.

**Published:** 1842-11-05

**Authors:** 


*A Case of Exophthalmia, with Œdema of the Conjunctiva, and Opacity of the Crystalline Lens in a Puerperal Woman.* By M. BLANDIN.—A woman, forty-one years old, was delivered, after a tedious labour, on December 3d, 1841. For fifteen days no unusual symptom occurred, but on the sixteenth and seventeenth day the patient was attacked by a violent shivering fit. On the eighteenth day, however, she returned from the hospital to her own home, and for some days afterwards suffered from febrile attacks, though they were no longer preceded by severe shivering. From the 25th of December the right eye began to project, the patient suffering little beyond a sense of weight in the head, principally in the supra orbitar region. Vision was at first unimpaired, but failed as the exophthalmia increased, and at last the patient became quite blind of that side. In this condition the patient applied to M. Blandin, at the Hôtel Dieu. There was then considerable prominence of the right eye, the conjunctiva of the globe was prominent, red, and swollen, and evidently infiltrated. The cornea was natural, the aqueous humour retained its transparency, and there was no evident change in the structure of the iris, but it had lost its contractility, and the eye was uninfluenced by exposure to a strong light. The crystalline lens appeared opaque, and of a shining, milk-white colour; the anterior membranes of the lens being in all probability the seat of the opacity. The volume of the globe was normal, the pain in the affected parts was inconsiderable, and no tumefaction existed of the parotid or cervical glands. The intellectual faculties were perfect and the general health was good.

In his remarks on the case M. Blandin offers some observations on the diagnosis of the affection. Some ramifications of the conjunctiva gave exit to a small quantity of pus from its inferior external portion. From that time the eye gradually retreated into the orbit, and from these circumstances M. Blandin concludes that there existed a small abscess behind the eye. The cause of the formation of this abscess is open to debate. It might be one of those purulent deposits occasionally met with in puerperal women. M. Blandin, however, regards it rather as the result of phlebitis, probably of the ophthalmic vein. He is likewise disposed to regard the affection as altogether analogous to *phlegmasia dolens*, in which disease the femoral vein becomes obliterated, just as here, in all probability, the ophthalmic vein was. On any other supposition the opacity of the capsule of the crystalline lens does not admit of explanation; while, in two other instances in which this lesion of the ophthalmic vein was discovered after death, precisely this condition of the crystalline lens had been noticed during the lifetime of the patient.—*Ibid, from Gazette des Hôpitaux.* Jan. 27, 1842.

